# Suicidal Acts Reported at a Teaching Hospital in Manipur

**DOI:** 10.4103/0970-0218.58400

**Published:** 2009-10

**Authors:** Vijaya Elangbam, Akoijam Brogen Singh, K Shantibala Devi, L Usharani Devi

**Affiliations:** Department of Community Medicine, Regional Institute of Medical Sciences, Imphal, Manipur - 795 004, India

## Introduction

Suicidal behavior ranges in degree from merely thinking about ending one's life, by developing a plan to commit suicide, obtaining the means to do so, attempting to kill oneself, and to finally carrying out the act (“completed suicide”). WHO defined ‘suicidal act’ as the injury with varying degrees of lethal intent and ‘suicide’ is defined as a suicidal act with fatal outcome.(1) The annual global mortality rate of suicide is about 14.5 per 100 000 people, which means that one suicidal death occurs about every 40 s. It is the thirteenth leading cause of death worldwide.(1) Suicide is under-reported by 20–100% according to prevailing beliefs and consequent negative notions and stigma attached to it in different cultures of the world.(2) There is dearth of data regarding suicide in Manipur. Hence, the present study was undertaken with the objective of determining the frequency of suicidal acts reported in casualty in RIMS hospital, Imphal and ascertain the association between suicidal acts and other variables.

## Materials and Methods

The study was a cross-sectional one, based on secondary data (casualty register) of the Regional Institute of Medical Sciences, Imphal. The participants included patients attending the casualty department of the Institute. All suicidal acts and suicidal cases reporting to casualty during a period of 1 year (1^st^ October 2004 – 30^th^ September 2005) were included. Data including age, sex time of suicidal act, date and means of committing suicide and other such details were extracted from the casualty register by using a performa. The outcome variables measured were frequency of suicidal acts by the total number of patients attending the casualty. Association between suicidal acts and the other study variables such as age, sex, and time of the suicidal act was also assessed. Statistical analysis was done using descriptive and chi-square tests. Permission to conduct this study was obtained from the institute authority.

## Results

All records were included for calculating the frequency of suicidal acts and 12 cases of suicidal acts were excluded from the subgroup analysis because of incomplete information. The total number of attendees recorded in the casualty register was 3953 and the number of suicidal act cases was 200. Frequency of suicidal acts was found to be 5.1 per 100 attendees. The frequency of suicidal act cases was found to be significantly higher in females than males (*χ*^2^= 5.30; *P*=0.012). Suicidal attempt by using poison was found to be the highest (85%), followed by hanging (5%).

The median age of the cases, excluding six cases with unknown age, was 25 years. The young adult age group (20–35 years) had the maximum number of suicidal acts and poisoning was the most common type of suicide in all age groups [Table 1]. Number of suicidal acts by poisoning was higher among females and during the summer season. The overall frequency of suicidal act cases was also highest in summer, but was found to be not significant. Most of the suicidal acts (80, 44.4%) were committed between 12 pm and 6 pm. Among the poisons, pesticides were the most common substances used for committing suicide by both the sexes (70/167) followed by disinfectants (33/167).

**Table 1:** Distribution of cases of suicidal acts by types and other variables

Variables	Type of suicide*					
	
	Hanging *n* (%)	Burn *n* (%)	Stab wound *n* (%)	Poison *n* (%)	Other forms *n* (%)	Total
Age (years)†						
10-19	1 (2.4)	1 (2.4)	1 (2.4)	39 (92.8)	0	42
20-35	8 (7.2)	2 (1.8)	2 (1.8)	91 (82.0)	8 (7.2)	111
36-59	0	1 (2.9)	2 (5.9)	30 (88.3)	1 (2.9)	34
≥60	0	0	0	5 (83.3)	1 (16.7)	6
Sex‡							
Male	7 (5.9)	3 (2.6)	5 (4.2)	95 (80.5)	8 (6.8)	118
Female	3 (3.8)	1 (1.3)	0	72 (92.3)	2 (2.6)	78
Season						
Spring	3 (6.8)	1 (2.3)	0	38 (86.4)	2 (4.5)	44
Summer	3 (5.5)	0	2 (3.5)	47 (85.5)	3 (5.5)	55
autumn	3 (5.5)	0	2 (3.5)	47 (85.5)	3 (5.5)	55
Winter	3 (6.0)	1 (2.0)	1 (2.0)	42 (84.0)	3 (6.0)	50
Time of suicide (h)§						
0–6	0	0	1 (5.9)	14 (82.3)	2 (11.8)	17
6–12	0	0	1 (4.0)	23 (92.0)	1 (4.0)	25
12–18	7 (8.7)	1 (1.3)	2 (2.5)	69 (86.2)	1 (1.3)	80
18–24	3 (4.6)	2 (3.1)	1 (1.5)	54 (83.1)	5 (7.7)	65

* 1 unknown type excluded

† Five cases of poisoning and one case of hanging were of unknown age

‡ Three unknown sex

§ one burn, one other forms, 10 poisoning cases were of unknown time

## Discussion

In this study, the frequency of suicidal acts was 5.1 per 100 attendees in the casualty department. Suicide rates for different parts of India ranged from 8.1 to 58.3/100000 people, which were from police records and were found to be under-reported.(3) However, the figures cannot be compared as the denominator used in our study was the total casualty attendees. Hence, further studies are needed in a community setting. Frequency of suicidal acts was significantly higher in females (7.2%) as compared with males (6.4%) in our study. While one study(4) found no significant difference of suicidal attempts among males and females, another study reported that suicide among males was more than females.(3) However, the gender differences seen in our study referred to suicidal attempts whereas the cited studies were on completed suicides. Hence interpretation of the findings should be made with caution. The predominance of females in attempted suicide may be due to the use of less lethal means of committing suicide and stronger social bonds, particularly motherhood.(5)

A maximum number of cases of suicidal acts were found in the young adult age group and poisoning was the most common method used. Similar findings were reported by Logaraj M(4) where the most common age group was 15-29 years, and poisoning and hanging were the most common methods. This may be due to easy availability of pesticides, as the majority of the people in Manipur engage mainly in agricultural activities. The younger age group is more ambitious and may be more prone to disillusionment and depression when faced with failure or resistance.

It was found that maximum number of cases of suicidal acts had occurred from 12 to 18 h followed by 18 to 24 h which was similar to the findings of other studies.(4) The higher occurrence of suicides during the latter part of the day and night time may be mainly due to diminished attention of others. According to this study, maximum number of cases of suicidal acts occurred in the summer. Other studies have reported maximum number of suicidal attempts at different times of the year such as February and March.(4)

Suicide by poisoning was the most common type of suicide in all age groups, pesticides being the most frequently used poison. So, selling of the pesticide should be monitored and regulated. As most of the suicides were attempted by the young adult age group, and them being in the most productive stage of life, there is a need for an integrated effort to check this. Creating awareness of the mass on suicide and increased involvement of expert counselors, specializing in adolescent problems, in preventing suicides are called for. The mental health program also needs to be effectively functional.
